# Running wheel access fails to resolve impaired sustainable health in mice feeding a high fat sucrose diet

**DOI:** 10.18632/aging.101857

**Published:** 2019-03-11

**Authors:** Aaffien C. Reijne, A. Talarovicova, Jolita Ciapaite, J.E. Bruggink, A. Bleeker, Albert K. Groen, Dirk-Jan Reijngoud, Barbara M. Bakker, Gertjan van Dijk

**Affiliations:** 1 Groningen Institute for Evolutionary Life Sciences, Dept of Behavioral Neuroscience, University of Groningen, Groningen, The Netherlands; 2 Department of Pediatrics, Center for Liver, Digestive and Metabolic Diseases, University of Groningen, University Medical Center Groningen, Groningen, The Netherlands; 3 Systems Biology Centre for Energy Metabolism and Ageing, University Medical Center Groningen, University of Groningen, Groningen, The Netherlands; 4 Center for Isotope Research, University of Groningen, Groningen, The Netherlands

**Keywords:** aging, activity, survival, diets, hormones

## Abstract

Diet and physical activity are thought to affect sustainable metabolic health and survival. To improve understanding, we studied survival of mice feeding a low-fat (LF) or high-saturated fat/high sugar (HFS) diet, each with or without free running wheel (RW) access. Additionally several endocrine and metabolic health indices were assessed at 6, 12, 18 and 24 months of age. As expected, HFS feeding left-shifted survival curve of mice compared to LF feeding, and this was associated with increased energy intake and increased (visceral/total) adiposity, liver triglycerides, and increased plasma cholesterol, corticosterone, HOMA-IR, and lowered adiponectin levels. Several of these health parameters improved (transiently) by RW access in HFS and LF fed mice (i.e., HOMA-IR, plasma corticosterone), others however deteriorated (transiently) by RW access only in HFS-fed mice (i.e., body adiposity, plasma resistin, and free cholesterol levels). Apart from these multiple and sometimes diverging health effects of RW access, RW access did not affect survival curves. Important to note, voluntary RW activity declined with age, but this effect was most pronounced in the HFS fed mice. These results thus challenge the hypothesis that voluntary wheel running can counteract HFS-induced deterioration of survival and metabolic health.

## Introduction

Nowadays people in modern societies live longer than ever before. The global share of individuals over 60 years increased from 9.2% in 1990 to 11.7% in 2013 and will continue to grow as a proportion of the world population, expected to reach 21.1% in 2050 [1]. The fact that people get older does not necessarily imply that the general population is becoming healthier. In fact, cardio-metabolic diseases associated with unhealthy habits are on the rise too and have a negative impact on sustainable health and lifespan [2].

During aging a gradual decline in muscle mass with a concomitant increase in fat mass and abdominal circumference takes place [3], often without changes in body weight or body mass index [4,5]. These changes could be a consequence as well as an underlying cause of the decline in functionality of the metabolic system [6–9]. An increase in adipose tissue particularly in the abdominal cavity is associated with insulin resistance and glucose intolerance, and may progress into diabetes [10–12]. Decreased lean mass can progress into sarcopenia, which results in frailty, poor immune function and impaired thermoregulation [13,14]. Feeding an obesogenic diet rich in saturated fat and refined sugars has been shown to reduce sustainable health in humans [15] as well in many animal models [16–18], and clearly opposes healthy aging [19]. Specifically, overconsumption of such a diet results in fat accumulation, increased energy expenditure, oxidative damage, cardiovascular disease, insulin resistance, and derangements in neuroendocrine functioning [20–23]. For this reason, eating diets low in saturated fat and fast sugars, but with high fibered carbohydrate content instead, is believed to be a key factor for healthy aging.

In addition to eating healthily, also maintenance of sufficient physical activity is important, since regular physical activity has been shown to attenuate the age-related decline in muscle mass and strength [24,25], to prevent fattening of the body [26], and to preserve metabolic functioning [27]. Thus, maintenance of physical activity appears to be an important asset to improved overall health in older individuals, and increases longevity [28–31]. These effects are most prominent when a high level of physical activity is commenced at young age and maintained towards old age [32].

Collectively data in literature suggest that the health-impeding effects of a diet high in saturated fat and refined sugars may be counteracted by physical activity and that this indirectly leads to increased longevity [28,30,31]. To our knowledge, however, there are no studies that specifically addressed this issue. For this reason, we investigated the effects of diet and exercise (and their interactions) on a number of metabolic health indices (triglyceride content in the liver, fat and lean masses, hormones, cholesterol and glucose homeostasis) and survival in male mice from weaning onwards exposed to a low-fat (LF) diet, or a high-saturated fat/high sugar (HFS) diet. Of each diet group, sub-cohorts had either free access to a running wheel from weaning onwards, or were kept under sedentary conditions. There are two important outcomes of our study. Firstly, mice fed a HFS diet had impaired sustainable health and longevity compared to those fed a LF diet, and this was not counteracted by voluntary wheel running. Secondly, voluntary wheel running declined with age, but much stronger in mice fed the HFS diet than those fed the LF diet. While the underlying neurobiological mechanisms of HFS-induced lowering of voluntary physical activity remains to be explored, our data imply that feeding a HFS diet causes both behavioral and physiological/metabolic changes that underpin impairment of sustainable health.

## RESULTS

### Life span

**Figure 1:**
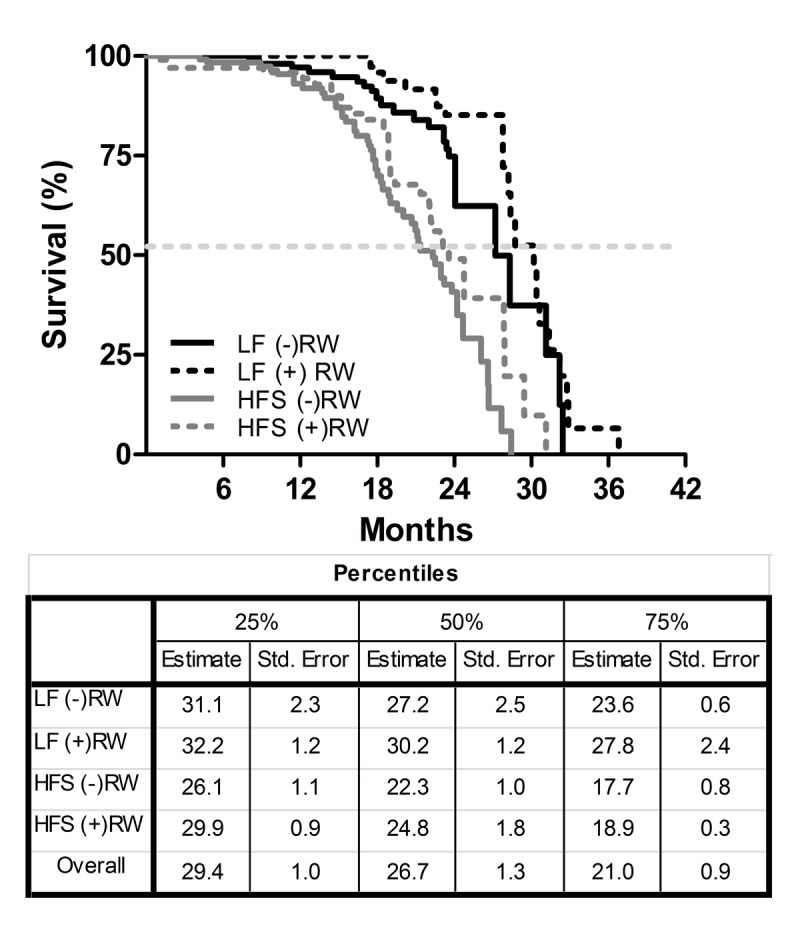
Kaplan-Meier survival curves of the four different experimental groups; two low fat (LF) diet groups, one sedentary (LF (-)RW) and one exercise group (LF (+)RW); and two high fat (HFS) groups, one sedentary (HFS (-)RW) and one exercise group (HFS (+)RW). Samples sizes were LF (-)RW n=119, LF (+)RW n=109, HFS (-)RW n=118, and HFS (+)RW n=100. There was a significant reduction in survival in sedentary mice on an HFS diet compared to LF diet (p<0.001, log-rank test) and there was a significant reduction in survival in exercising mice on a HFS diet compared to LF diet (p<0.01, log-rank test). The table below the figure indicates the three different percentiles of the four different experimental groups, the 50% percentile is also indicated with the dotted line in the figure.

The survival characteristics for the four groups are shown in Figure 1. There was a significant effect of diet on life span under sedentary as well as running wheel conditions (LF (-)RW vs HFS (-)RW p<0.001; LF (+)RW vs HFS (+)RW p<0.001). Running wheel availability tended to cause a right shift of the survival curves under both diet conditions (i.e., LF (-)RW vs LF (+)RW p=0.072 and HFS (-)RW vs HFS (+)RW p=0.081). This indicates that eating a HFS diet decreased survival independent of activity status.

The medians of the four different groups differed from each other (K-independent samples median test p<0.05). After testing the separate groups (Mann-Whitney Test) again a diet effect was found; the medians of LF (-)RW and LF (+)RW differed significantly from medians of HFS (-)RW (p<0.01) and HFS (+)RW (p<0.01) groups, respectively.

In Table 1 the death causes, including numbers and percentages are indicated. Mice were mostly sacrificed because of sudden severe body weight loss, in combination with a general bad appearance and wound scratching, mainly under HFS diet conditions. There was no significant association between experimental group and cause of death (χ^2^ Likelihood Ratio p=0.282).

**Table 1:** Reasons and numbers of sacrifices during this study.

Group	Severe weight loss / bad appearance	Scratching wounds	Anal problems	Eye infection	Cancer	Locomotion problems	Tilted head	Teeth/ breathing problems	Experimental reasons	Death in cage	Total n
LF (-)RW (n=119)	7	4	1		1		1		1	8	n=23
LF (+)RW (n=109)	6	2		2	1		1	1		8	n=21
HFS (-)RW (n=118)	10	12	4	1			1	3	2	7	n=40
HFS (+)RW (n=100)	14	5		1	1	4		1	2	4	n=32
Total n	n=37	n=23	n=5	n=4	n=3	n=4	n=3	n=5	n=5	n=27	n=116

On the different rows the different experimental groups are indicated, in the different columns the causes of death. Planned sacrifices (6, 12, 18 and 24 months) are excluded from these results.

### Animal characteristics

**Figure 2:**
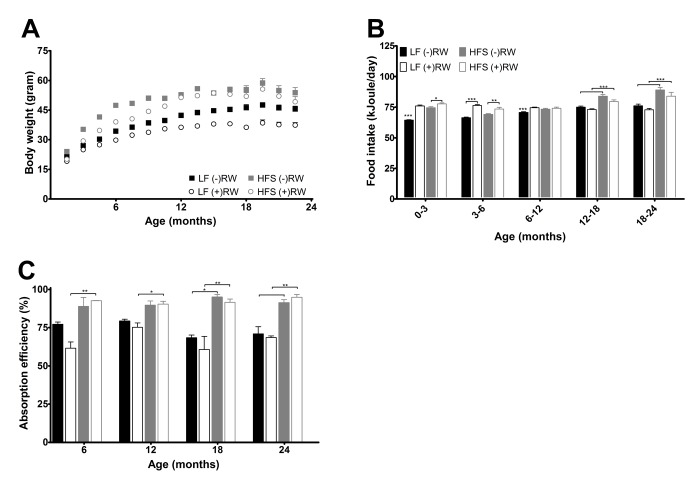
(**A)** Body weight development measured over their life time and indicated every 1.5 months. There was a significant reduction of body weight in mice on a LF diet (p<0.001), in mice with access to a running wheel (p<0.001), and an increase in body weight with age (p<0.001). Data are averages from n=18-104 mice per group; ± SEM. (**B**) Average 3 or 6 months energy intake. Data are averages from n=18-104 mice per group; ± SEM *p<0.05 **p<0.01 ***p<0.001 per time point analysis (Bonferonni corrected) (**C**) Absorption efficiencies for the four experimental groups at the four different time points. Absorption efficiencies were higher in mice on HFS diet compared to mice on a LF diet. Data are averages from n=2-3 mice per group; ± SEM *p<0.05, **p<0.01 per time point analysis (Bonferonni corrected).

**Figure 3:**
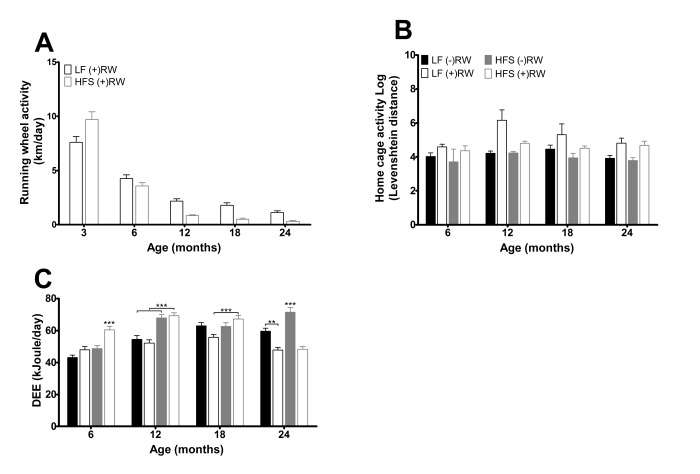
(**A**) Running wheel activity for the two different running wheel groups at five different time points. Activity was higher in mice on a LF diet (p<0.001) and activity decreased with age (p<0.001). Data are averages from n=15-50 mice per group; ± SEM. (**B**) Home cage activity for the four different groups at the four different time points. Activity was higher in mice with access to a running wheel (p<0.001) and was higher in mice on a LF diet (p<0.001). Data are averages from n=3-12 mice per group; ± SEM. (**C**) Daily energy expenditure (DEE) for the four different groups at the four different time points. Data are averages from n=7-8 mice per group; ± SEM **p<0.01, ***p<0.001 per time point analysis (Bonferonni corrected).

There were no differences in body weight at weaning (data not shown). Mice eating a HFS diet had overall a higher body weight compared to mice fed a LF diet (p<0.001), and (+)RW mice had overall a lower body weight compared to (-)RW mice (p<0.001) (Figure 2A). (+)RW mice on a HFS diet had a lower body weight than the (-)RW mice on a HFS diet, although from 12 months onwards the differences became smaller. This latter part was also the time frame where the running wheel activity of the HFS (+)RW mice became very low compared to the LF (+)RW mice (Figure 3A). Over the total 24 months energy intake changed over time (Figure 2B, p<0.001), it was higher in HFS mice compared to LF mice (p<0.001), and there was a running wheel effect (p<0.001) which altered over time (i.e., at young age (+)RW mice ate more than the (-)RW groups).

At four different time points (6, 12, 18 and 24 months) feces were collected over a period of 48 hours and its energy content was measured (Figure 2C). Absorbed energy was calculated from differences between energy intake and fecal energy content. Overall the HFS diet was absorbed more efficiently than the LF diet (p<0.001). In addition, mice with access to a running wheel on a HFS diet were more efficient, while mice with access to a running wheel on a LF diet were less efficient compared to their sedentary counterparts (p<0.05).

At three months of age the HFS (+)RW mice had higher running wheel activity than LF (+)RW mice, but after that their running wheel activity deteriorated and they became less active in their running wheels than the LF (+)RW mice (Figure 3A, p<0.001). Home cage activity, excluding running wheel activity, assessed by IdTracker revealed an overall running wheel, diet and age effect (Figure 3B), indicating that home cage activity was higher in both LF compared to HFS groups and both running wheel groups compared to sedentary groups.

The daily energy expenditure (DEE) results assessed with the doubly labeled water method are depicted in Figure 3C. Overall DEE was higher in mice feeding a HFS compared to mice on a LF diet (p<0.001). The overall effects of age (p<0.001) and running wheel (p<0.05) indicate that the mice with access to a running wheel have a higher DEE at young age a while at older age it is lower (time*running wheel p<0.001) compared to sedentary mice, and overall DEE increased with age (p<0.001). Note that DEE is measured in a 48 hours interval, at a certain age, while food intake is averaged over a 3 or 6 months interval.

**Figure 4:**
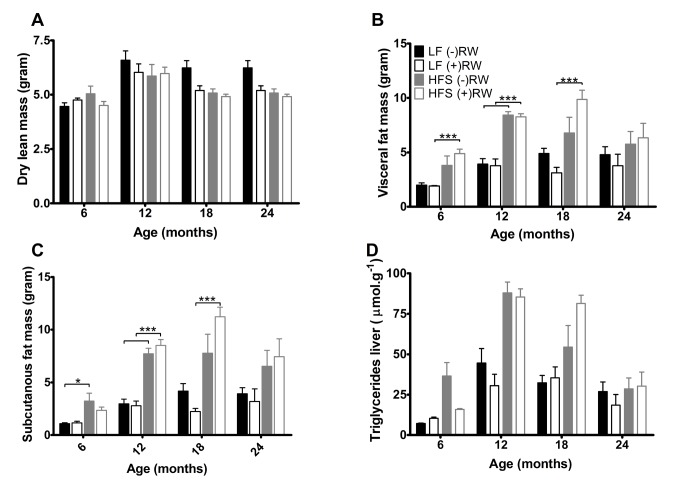
(**A**) Dry lean mass, was higher in sedentary compared to running wheel mice (p<0.001). Data are averages from n=7-8 mice per group; + SEM. (**B**) Visceral fat mass at the four different ages. Data are averages from n=7-8 mice per group; + SEM ***p<0.001 (**C**) Subcutaneous fat mass at the four different ages. Data are averages from n-7-8 mice per group; ± SEM ***p<0.001, *p<0.05 (**D**) Liver triglycerides, increased with age (p<0.001) and on a HFS diet (p<0.001). Data are averages from n=7-8 mice per group; + SEM.

At 6, 12, 18 and 24 months of age mice were sacrificed and body composition analysis was performed (Figure 4A), showing that dry lean mass was lower in running wheel groups (p<0.05) mostly due to the LF groups.

Total white fat (not shown), subcutaneous plus visceral fat, was higher in the HFS mice compared to LF mice at 6 and 12 months of age and this effect was not counteracted by giving these mice access to a running wheel. At 18 months of age there was an counteraction effect in the LF group resulting in less fat mass, while the HFS (+)RW mice became fatter compared to HFS (-)RW mice. Liver triglycerides increased on a HFS diet independently of RW access (Figure 4D).

**Figure 5:**
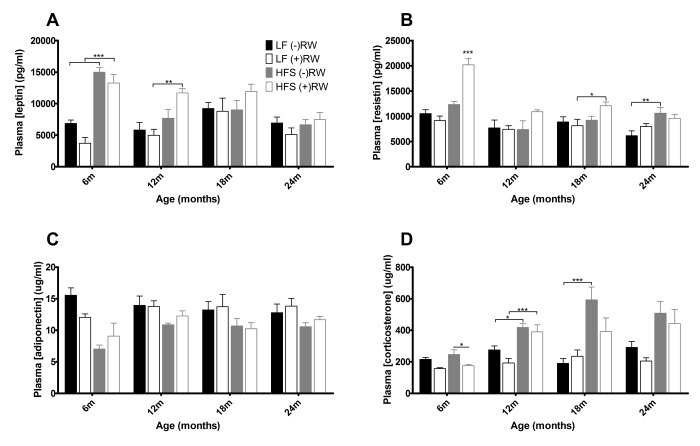
Blood plasma results. (**A**) leptin was at young age higher in HFS diet groups (**B**) resistin was increased in mice on a HFS diet with access to a running wheel (**C**) adiponectin was higher in mice on a LF diet (**D**) cortisol was higher in mice on a HFS diet. Data are averages from n=6-8 mice per group; + SEM. *p<0.05, **p<0.01, ***p<0.001 (per time point analysis (Bonferonni corrected)).

During sacrifice, blood samples were taken for further analyses. Figure 5 depicts the results of several hormone analyses. At young age leptin levels (Figure 5A) were higher in HFS mice compared to LF diet mice, this effect disappeared at 18 and 24 months of age. Resistin (Figure 5B) was higher in mice on a HFS diet with access to a running wheel, although this effect disappeared at 24 months. Adiponectin (Figure 5C) was higher in mice on a LF diet compared to mice on a HFS diet and corticosterone (Figure 5D) was higher in mice on a HFS diet compared to mice on a LF diet.

**Figure 6:**
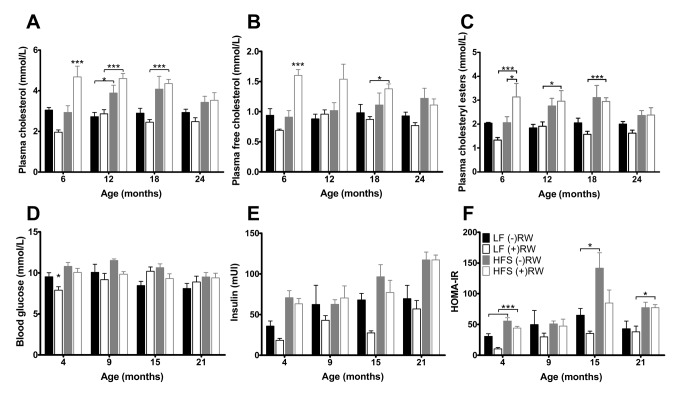
Glucose and cholesterol blood plasma results. (**A**) Blood glucose after 6 hours of fasting; (**B**) Blood insulin after 6 hours of fasting, increased with age and eating a HFS diet and decreased with having access to a running wheel; (**C**) HOMA-IR index was improved in mice on a LF diet; (**D**) Total plasma cholesterol was higher in mice on a HFS diet; (**E**) free plasma cholesterol was highest in mice on a HFS diet in combination with access to a running wheel (**F**) plasma cholesteryl esters was higher in mice on a HFS diet. Data are averages from n=6-8 mice per group; + SEM. *p<0.05, **p<0.01, ***p<0.001 (per time point analysis (Bonferonni corrected)).

Plasma cholesterol (Figure 6A) was (except at 6 months of age) higher in mice on a HF diet compared to mice on a LF. Plasma free cholesterol was highest in mice on a HF diet with access to a RW, at 24 months of age the RW effect was absent (Figure 6B). Also, the plasma cholesteryl esters (Figure 6C) were higher in mice on a HF diet, having access to a RW increased the esters at 6 months of age, after that this effect was absent.

At 4, 9, 15 and 21 months of age blood glucose samples were taken (Figure 6 D-F). After 6 hours of fasting insulin resistance assessed according to the homeostatic model (HOMA-IR) was decreased in mice with access to RW compared to mice on the same diet without RW access indicating that voluntary activity increases insulin sensitivity. At 21 months of age, this effect was absent.

To check whether certain measurable factors could indicate the life span of the animals, several correlations were made. Overall there was no correlation (tested with Pearson) between life span and maximum or minimum body weight, running wheel activity (while all mice were still alive) or running wheel at a certain time point (data not shown).

## DISCUSSION

The main outcome of the present study is that continuous feeding (i.e., from weaning onwards) a diet rich in saturated fats and sucrose (HFS) impairs longevity in mice, as indicated by a left shift of the survival curve in that group of mice compared to that of mice feeding a healthy fibered low fat (LF) diet and this effect is not prevented/rescued by offering a running wheel in which mice could run voluntarily. The health undermining effects of HFS feeding in the present study is consistent with several other studies showing adverse health consequences of ‘western-style’ diets in mice [16–18], and other animals [33,34]. Running wheel availability improved some metabolic health indices in mice feeding the HFS diet, but also worsened a few of them. These findings highlight the importance of eating a healthy diet for maintenance of sustainable metabolic health, and that solely offering ways to increase ‘voluntary’ physical activity is not necessarily beneficial for improving metabolic health in the face of eating an unhealthy diet.

Small daily overconsumption relative to expenditure eventually leads to increases in fat mass and/or -depending on developmental phase - lean mass [35,36]. It is clear from the comparison of the charts of the intake and the daily energy expenditure (DEE) data that the mismatch between the two was largest around 6 months for both the HFS and LF fed groups, when the animals were still building up lean mass (Figures 2B and 3C). However, when the latter process ended, a pronounced increase in adipose tissue storage became apparent particularly in the HFS fed mice (Figures 4B and C). We can describe three processes that may have played a role in this mechanism. First of all, caloric intake went up in the mice on the HFS diet after 12 months compared to the LF fed mice, whereas their energy expenditure stabilized. The higher absorption efficiency of the HFS diet probably contributed also to this effect, although not necessarily only after 12 months. Secondly, at 12 months of age and later, plasma levels of corticosterone were increased in the HFS fed animals compared to the LF animals. Increased glucocorticoid action impairs leptin sensitivity in brain regions involved in energy balance regulation [37], which may have contributed to the increased caloric intake in the HFS fed mice [38]. Additionally, increased glucocorticoid action causes insulin resistance [39], which explains also the increased HOMA-IR index (i.e., a proxy for insulin resistance [40]) in the HFS fed mice. Of interest is the relative decline in plasma leptin levels over time that we observed in the HFS fed mice, which we did not expect when glucocorticoid increases [37]. At this moment, we do not have an explanation for the reduction in plasma leptin levels over time, but it would likely have contributed to unleash mechanisms that would normally be inhibited by leptin [38], among others an increase in caloric intake and adiposity stores. This lowering of plasma leptin, combined with the increase in glucocorticoid, as well as the profound increase in visceral adiposity could all contribute to elevation of triglyceride storage in the liver [41–43], which together with insulin resistance, are hallmarks of the metabolic syndrome [44]. Increased fat overflow from visceral depots has been described to stimulate hypothalamic-pituitary-adrenal (HPA) axis activity, which could become a self-perpetuating cycle [45]. Hyperactivity of the HPA-axis results in increased corticosteroid levels, which again might lead to increased lipogenesis and decreased free fatty acids beta-oxidation. The lower levels of plasma adiponectin in the HFS fed mice compared to LF mice are also in line with this. In this respect, we also analyzed circulating cytokine levels (tumor necrosis factor alpha (TNFalpha), interleukin(IL)-1 and IL-6, data not shown), but these were not significantly altered over age, nor between running wheel/sedentary and/or diet groups. This observation is consistent with data describing only increases in circulating cytokines during inflammatory insults, like experimental exposure to lipopolysaccharide [46].

Another comparison in the present study can be made between sedentary and running wheel groups of mice. At 6 months, access to running wheels improved HOMA-IR and lowered plasma corticosterone irrespective of diet. The HOMA-IR results are in line with several papers highlighting the positive role of muscular activity on glucose homeostasis [47,48]. Literature on chronic effects of running wheel activity on plasma corticosterone is not consistent, several studies indicate increased levels [49,50], decreased levels [51] and no change in corticosterone levels [52,53]. It is of note that the HOMA-IR and lowered plasma corticosterone effects were not related to alterations in body fat content as has been shown in other studies [54,55]. Besides the health-improving effects of wheel running behavior, there were however also some indications that running wheel access had some potentially negative effects, specifically in the HFS fed mice, such as the temporally increased levels of plasma resistin, cholesterol, and body adiposity. The effect of wheel running to increase resistin levels in our mice may be homologous to the observation that elevated resistin levels are found in human elite athletes [56] and subjects performing an aerobic exercise training program [57]. The underlying causes are poorly understood, but - at least in the present study - they were not related to changes in the adipokines leptin and adiponectin (Figure 5A and C), nor to circulating glucocorticoids (Figure 5D), but they did depend on feeding the HFS diet. Resistin is known to increase very low-density lipoprotein (VLDL) and low-density lipoprotein (LDL) cholesterol production [58,59], and this mechanism could explain the elevated plasma levels of (total) cholesterol and cholesteryl esters that we observed in the HFS fed mice with access to running wheels. Although these effects at young age might have been adaptive (e.g., for sustaining high levels of physical activity), they may have had deleterious side effects when physical activity levels sharply reduced when animals became older. It may be speculated that the presumed resistin-cholesterol link counteracted the initial health-promoting effects of running wheel activity when the mice aged. Indeed, we found increased levels of visceral and total adiposity as well as liver triglyceride accumulation in the HFS mice with access to running wheels compared to their sedentary controls when the animals aged (Figure 4). It may be speculated that these processes are inherently linked to declining liver health, as resistin has been identified as an intra-hepatic cytokine as well [60]. Important for consideration of these results is the finding that running wheel activity declined rapidly with aging, but this effect was most outspoken in the group of mice feeding the HFS diet. The largest decline in the HFS fed mice was observed from 6 to 12 months, which preceded the time at which differences in survival curves started to become apparent between HFS and LF fed mice. The neurobiological mechanism underlying reduced wheel running behavior remains elusive, but may have resulted from reduced activity of brain D2 receptor functioning [61]. The latter is long known to occur with aging [62], as well as with development of obesity and associated co-morbidities [61].

From our data it is difficult to attribute differences in survival to specific changes in metabolic, endocrine and behavioral effects of diet and/or physical activity. In addition, our data suffers, like any other aging study, from survivor bias selecting those animals from 12 months of age onwards that did not die spontaneously. The fact that the HFS fed mice with access to running wheels presented a transient increase in visceral fat and hepatic triglyceride content around 18 months, but not thereafter is probably an example of such a survivor effect. We did not find markers of energy balance and fuel homeostasis in animals alive that explained some of the variation in life span beyond diet, and we are not aware of any study that did, besides the well-known examples like high adiponectin levels [63], Sirt copy numbers [64], inhibited mammalian target of rapamycin (mTOR) [65], and growth hormone/insulin-like growth factor (IGF) alterations [66].

Physical activity, in captive animals mostly via voluntary wheel running, is studied for many health promoting effects including preventing cancer [67,68], obesity and diabetes [26,27,69–71], learning and memory [72,73], and so on. Indirectly these health benefits might contribute to healthy aging and improved survival. Studies focusing on the effect of exercise on aging gave contradicting results [32]. Reasons for this could be the health status of the animals [29,74], various background factors like nutritional status [75], the starting point of the exercise paradigm [32], and the type/intensity of the exercise [76]. In our study, the exercise intensity of voluntary wheel running can be considered moderate and non-exhaustive [77]. In fact, Koteja and colleagues [76] have calculated that mice running comparable distances as the mice in our study at 6 months of age (i.e., ~4000 m/day) use only ~4% of their daily energy budget for running. High intensity exercise can in our view only be found in mice that are forced to run for instance on a treadmill [78,79] or when running is connected to highly rewarding stimuli [80]. Provided that energy efficiency of running is maintained over time, the data collectively would suggest a further decline in the excess daily energy expenditure allotted to running wheel behavior in our mice at 12 months or older [81]. It is of note that the availability of a running wheel did not impair home cage activity compared to mice without running wheels, and these levels remained rather stable throughout the 24-months’ measuring period. It may therefore be speculated that the reduced intention of running voluntarily in a wheel is a better indicator for decline of metabolic functioning than home cage activity per sé.

To our knowledge, a study hypothesizing that the worsening effect of a HFS diet could be counteracted by voluntary exercise has not been performed. In our study this hypothesis is rejected. At 24 months, however, there was a trend (p= 0.089) in survival difference between the HFS (-)RW and HFS (+)RW, suggesting that if we would have continued the study for a longer period this might have resulted in a significant difference between these two groups. This does not seem to happen in mice on a LF diet. The fact that the decline of voluntary wheel running behavior occurred most strongest in mice on the HFS diet raises the question what would happen if the activity level was kept at that of the mice at 6 months of age in both groups throughout their lives. Whether this would result in life span increasing effects because of the healthy exercise component or in life span decreasing effects because of the potentially unhealthy chronic stress component of forced behavior [82] needs to be studied. In conclusion, continuous feeding a diet rich in saturated fats and sucrose impairs longevity in mice and continuous access to a running wheel did not significantly rescue this effect. It is difficult to attribute the differences in survival to specific changes in metabolic, endocrine and behavioral effects of diet and/or physical activity.

## MATERIALS AND METHODS

### Animals and experimental protocol

Male C57BL6/JOlaHsd mice (n=449 at start of the study) (Harlan Netherlands BV, Horst, The Netherlands) were housed individually from weaning onwards on a 12hr:12hr light:dark cycle in a temperature-controlled environment (22+1 °C) with *ad libitum* access to standard lab chow (LF, 6% fat, AMII 2141, 19.1 kJoule/gram, HopeFarms BV, Woerden, NL) or high fat diet with lard and refined sugars (HFS, 45% fat, 4031.09, 17.5 kJoule/gram, HopeFarms BV, Woerden, NL) and water. Each diet group was further subdivided into groups with continuous voluntary access to a running wheel ((+)RW) or without access ((-)RW). These and all other methods were approved by, and are in agreement with the regulations of the Institutional Animal Use and Care Committee of the University of Groningen. These regulations are consistent with the guidelines for the care and use of laboratory animals as described by the U.S. National Institutes of Health.

Sample sizes at the beginning of the study were LF (-)RW n=119, LF (+)RW n=109, HFS (-)RW n=118, and HFS (+)RW n=100, these numbers declined during the study because of planned sacrifice (n=64). We recorded survival of the mice that were not planned to be sacrificed.

Regulations of the Institutional Animal Use and Care Committee of the University of Groningen require that animals should be terminated when they show signs of poor general health, including sudden and severe weight loss (>20% within days) in combination with either bad appearance, severe scratching, eye/teeth/anal problems, evident cancer, vestibular deficit/tilted head. These indicators are predictors of death within a few days and left-shifted survival curves (i.e., compared to the situation in which animals would die naturally) a few days to the most.

During this study, body weight and energy intake were assessed every 3 weeks. At 6, 12, 18 and 24 months of age, subgroups of mice were sacrificed to analyze body composition, plasma contents and triglyceride content of liver.

### Running wheel activity

Voluntary wheel running activity was recorded throughout the lives of the mice. The passing of a small magnet attached to a running wheel (and counterbalanced by an equal weight on the opposite side) across the sensor on the cage signaled a wheel revolution. These revolutions were collected continuously and stored in minute bins by a Circadian Activity Monitor System (CAMS, by H.M. Cooper, JA Cooper, INSERM U846, Department of Chronobiology, Bron, France). Initial inspection of the data was done by importing the data into a custom made excel macro package (ActoView, C.K. Mulder, Department of Molecular Neurobiology, Groningen, Netherlands). Initial imports condensed the data to 60-minute bins, allowing easy visual inspection of long-term recordings. Monthly activity measures were calculated for a month of age ‘*m*’, by averaging overall daily activity from postnatal day 30*m - 14 to 30*m + 14. The use of this large time period improved the validity of the resulting averages and to eliminate confounding effects of e.g.: daily variation in individual wheel running activity; weekdays vs. weekends; (husbandry) activities in the animal rooms (recordings were briefly paused during husbandry activities). The numbers of wheel revolutions per time period were converted to the metric system, to provide a more meaningful measure.

### Home cage activity

A subset of mice in their cages at 6, 12, 18 and 24 months of age was video-recorded for 24 hours to assess home cage activity in a 2D plane (i.e., from the long side of the cage), outside their running wheels. Locomotor behavior was analyzed using IdTracker, which is a freely available software package [83]. Because this method was developed for analysis of locomotor behavior of individuals within a group, we used it initially to assess multiple mouse cages at once. Its methodology is distinct from previous approaches in that idTracker extracts from the video a signature or fingerprint for each individual. These fingerprints were used to identify individuals in each frame, keeping the correct identities. Trajectories were then obtained by joining the centers of the labeled individuals. The method claims that it does not suffer from propagation of errors, giving reliably correct identities even for long videos and any complexity of crossings [83]. To avoid potential overlap of animals, we decided to frame-grab each mouse separately for behavioral analysis. Behavioral analysis with IdTracker software was verified with Ethovision [84] - which is a validated behavioral analysis software package – in a few animals and yielded comparable results.

### Doubly labeled water measurements

Mice were weighed to the nearest 0.1 gram and injected intraperitoneal with ~0.12 g of enriched water (66.6% ^18^O, 33.3% ^2^H). The syringe carrying the doubly labeled water was weighed to the nearest 0.0001 gram before and after injection. After two hours of equilibration, mice were bled at the tail tip, and two initial blood samples (20 μL) were collected in duplicate glass capillary tubes, which were immediately flame-sealed with an isobutene torch. A final blood sample was taken 48 hours after the collection of the initial blood sample. These measurements were performed under the different housing conditions of the experimental groups.

Determinations of ^2^H/^1^H and ^18^O/^16^O ratios in blood samples were performed at the Center for Isotope Research (University of Groningen, Energy and Sustainability Research Institute Groningen, The Netherlands). A detailed description of the analytical procedures followed in our laboratory has been published [85].

### Bomb calorimetry measurements

During the doubly labeled water measurements (48 hours), feces of animals were collected from the saw-dust bedding and weighed. The caloric content of the feces was measured by bomb calorimetry. A known amount of benzoic acid (energy content of 26.44 kJ/g) was combusted in the bomb calorimeter. Following samples of the feces were combusted and compared to the heat production of the reference to determine the energy content of the samples. These data, combined with the assessed energy intake, enabled us to estimate the absorption efficiency.

### Determination of body composition

Mice sacrificed at 6 (n=16, divided in two different groups), 12 (n=16, divided in two different groups), 18 (n=16, divided in two different groups), and 24 (n=16, divided in two different groups) months of age were processed for determination of body composition. Dry and dry lean organ masses were determined by drying organs to a constant mass for 14 days at 60 °C followed by fat extraction with petroleum ether (Boom BC, Meppel, The Netherlands) in a custom made soxhlet apparatus. Pieces of deeply frozen liver were weighed. For triglyceride determination 10% homogenates (w/v) were prepared in ice-cold PBS (pH 7.4). Lipids were extracted according to Bligh and Dyer [86]. Triglyceride content was determined with a commercial kit (Roche Diagnostics, Mannheim, Germany) according to manufacturer’s recommendations. N=7-8 per group.

### Plasma analysis

At 4, 9, 15 and 21 months of age mice were fasted for 6 hours. Blood glucose concentrations, sampled by tail bleeding, were measured using a EuroFlash meter (Lifescan Benelux, Beerse, Belgium). In addition to the blood drop needed for glucose concentrations, blood samples were drawn by tail bleeding into heparinized tubes. This blood was centrifuged (4000 g for 10 min) and plasma was stored at -80°C. Plasma insulin values were determined using Enzyme Linked ImmunoSorbent Assay (ELISA, ALPCO Diagnostics, Salem, United States) and HOMA-IR was calculated according to (IR – (fasting insulin in mU/L x fasting glucose in mM)/14.1) [87].

At the different ages of sacrifice (6, 12, 18 and 24 months) a blood sample was taken by heart puncture. The mice were not fasted prior to blood sampling and all mice were under anesthesia when the blood sample was taken. Blood was collected in tubes with anti-coagulant (EDTA). Samples were spun down at 26000g for 15 minutes at 4°C. Plasma was collected and stored at -80°C until analysis of concentrations of various hormones.

Plasma concentrations of leptin and resistin were measured using Multiplex Biomarker Immunoassays for Luminex xMAP technology (Millipore, Billerica, MA, USA; cat. No. MMHMAG-44K). Commercially available kits were used to measure plasma levels of adiponectin (Millipore, Billerica, MA, USA; cat. No. EZMADP-60K), corticosterone (MP biomedicals, LLC, Orangeburg, NY, cat. No. 07-120102), total and free cholesterol (Diasys, Holzheim, Germany). Cholesteryl esters were calculated from the difference between total cholesterol and free cholesterol.

### Statistical analysis

Survival curves were prepared using the product limit method of Kaplan and Meier [88]. Statistical differences between the curves were tested for significance using the log-rank test. A K-independent samples median test was used to test the four different medians of the curves together, after that a Mann-Whitney test was used to test if the medians of the separate groups differed from each other.

All data (with the exception of the survival curves) is expressed as averages + standard error of the mean and were tested for normal distribution. To check whether there was a significant association between cause of death and experimental group a chi square analysis was performed.

For body weight, food intake and running wheel activity the overall statistical significance of age and treatment (diet and/or running wheel activity) were assessed using a diagonal mixed model analyses. If significant effects were found, a Bonferroni corrected mixed model analysis per time point was performed.

For home cage activity, daily energy expenditure, absorption efficiency, dry lean mass, liver triglycerides, fat mass of the mice, hormones, cholesterol- and glucose data the statistical significance of age and treatment (diet and/or running wheel activity) effects were assessed using two-way analysis of variance (ANOVA) with three between-subjects factors (age, diet and running wheel activity). Only if an interaction term between the factors was found to be significant, the effect of each factor was analyzed separately using Tukey post-hoc test.

All analyses were performed with SPSS 23.0 (SPSS Inc., Chicago, IL, USA). Level of statistical significance was set at p<0.05.
